# Spontaneous bleeding of an Abrikossoff's tumor - a case report

**DOI:** 10.1186/1749-8090-4-57

**Published:** 2009-10-28

**Authors:** Philipp Honigmann, Alexander Walz, Christian Bussmann, Bruno Lerf

**Affiliations:** 1 Surgical Department, Cantonal Hospital Zug, Switzerland; 2 Department of Internal Medicine, Cantonal Hospital Zug, Switzerland; 3 Pathological Department, Cantonal Hospital Lucerne, Switzerland

## Abstract

Abrikossoff tumors are a rare tumor entity. The complication of a hemothorax has not been described in the literature so far. A 24-year-old patient presented with repeated hemoptysis and right thoracic pain. The initial CT-scan revealed a solid tumor mass in the right lower bronchus. After further diagnostics, the patient was discharged and surgical intervention was planned. He was readmitted 4 days after discharge with a spontaneous hemothorax. After the right lower lobectomy and an uneventful course the patient recovered well.

## Case presentation

Our 24-year-old non-smoking male patient presented with repeated hemoptysis in May 2008 with 4 days of concomitant right thoracic pain which intensified while breathing. During holidays in his home country, this Cuban patient suffered from a cold with fever and a strong cough. The strong dry cough persisted after recovery from the cold. The patient did not report any loss of weight.

**Figure 1:**
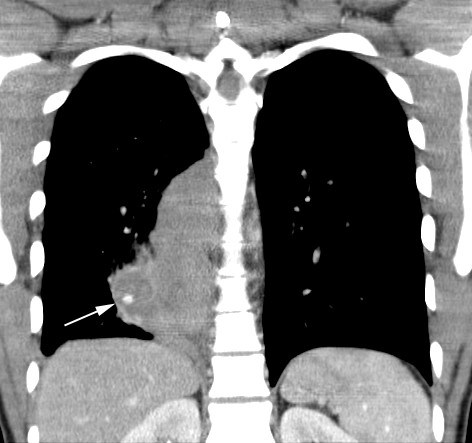
**CT-reconstruction**.

**Figure 2:**
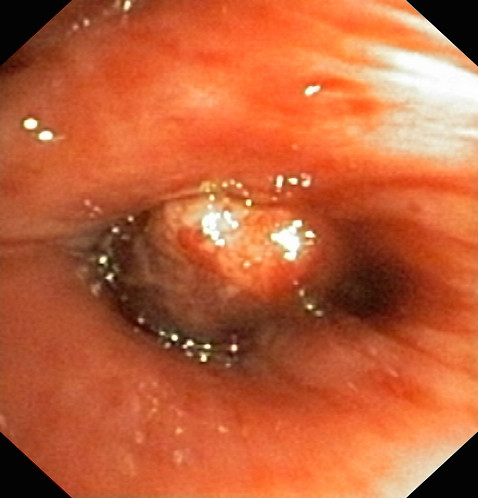
**Tumor mass (bronchoscopy)**.

The initial CT scan of the thorax showed a 12 × 4 cm solid mass paravertebral right in the lower thorax without any signs of metastases (Figure 1). The bronchoscopy (Figure 2) with non-bleeding biopsy revealed a mass of the lower right bronchus which histologically and immunohistologically provided evidence of a granular cell or Abrikossoff tumor [1]. The bronchial lavage which followed was negative for malignant cells. The patient was discharged and surgical intervention was planned.

**Figure 3:**
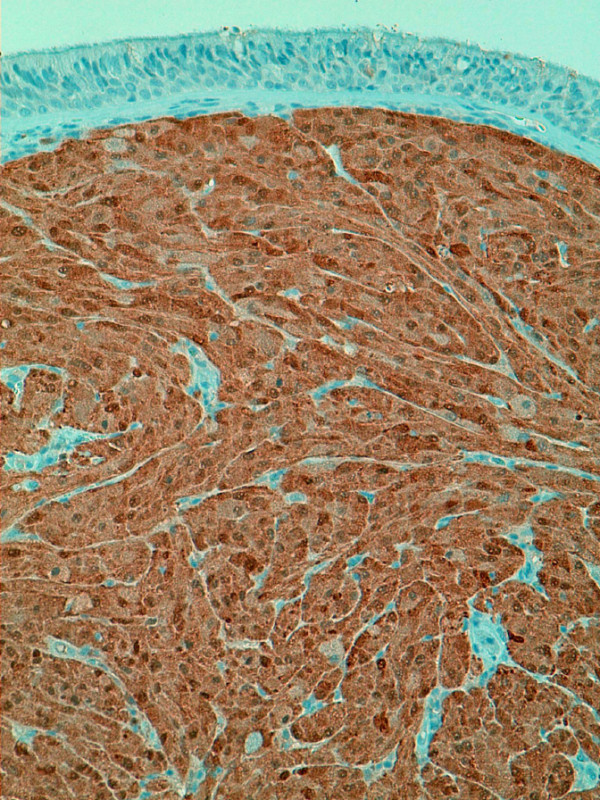
**Immunohistological image (zoom 20 ×; S100)**.

Four days after discharge a spontaneous hemothorax developed. The patient needed to be readmitted and the hemothorax was drained. No malignant cells were detected in the cytological examination of the drained liquid. After an uneventful course and decreasing of the hematoma, the tumor was excised by performing a lower right lobectomy 6 months after the initial admission. The final histological examination confirmed a peribronchial and infiltrating S100 positive tumor supporting the Schwann cell origin theory with very low growth rate of 2% and a size of 15 mm (Figure 3).

About 130 cases of pulmonary occurrence of Abrikossoff's tumor have been described in the literature up until now. Van der Maten et al. [2] reported an incidence of this mostly benign and slow-growing tumor in the tracheobronchial system in the Netherlands of 2:100,000. In this retrospective case series, the upper tracheobronchial system was more frequently affected than the lower part, and 65% of the patients were smokers. Valenstein [3] reported a more frequent occurrence on the right than on the left side, and most commonly with a cough as the presenting symptom. This kind of tumor can occur anywhere in the body, but mainly in the head and neck region, mostly intraoral [4-7]. Other localizations are the skin, thoracic region, breast and GI-tract [8,9]. Only 10% are located in the pulmonary system and of these, 25% are multiple occurrences. Deavers [10] presented a slight trend for a predilection of dark-skinned patients. He also reported on the infiltrative nature of this tumor and described a peribronchial tissue extension of 48% which often makes it impossible to excise the tumor bronchoscopically. Daniel et al. [11] reported that tumors with a diameter of 8 mm or greater are likely to invade the full-thickness bronchial wall, with infiltration into the peribronchial tissue. They recommend a lobectomy or pneumonectomy for the treatment of bronchial tumors with extensive destruction of distal tissue. If there is no extensive distal suppuration or tissue destruction the tumors can be excised bronchoscopically as long as they are less than 8 mm in diameter. Bronchoscopical treatment of larger tumors is associated with a significant increase in the recurrence rate. In addition, the hemorrhage rate is also increased [12,13].

Our patient recovered fully from the surgical intervention and presented in very good condition during follow-up.

## Conclusion

Vascular arrosions of this tumor entity have not been described in the literature so far. The occurrence of a hemothorax is a rare complication but one which has to be kept in mind by the treating surgeon.

## Competing interests

The authors declare that they have no competing interests.

## Authors' contributions

PH is the author of the manuscript, AW was the initial doctor in charge, CB is the pathologist, BL performed the lobectomy as head of surgical department. All authors have read and approved the final version of this manuscript.

## Consent

Written informed consent was obtained from the patient for this publication including any accompanying images. A copy of the signed consent is available for review by the Editor-in-Chief of this journal.
